# Strengthening regional surveillance: MenMap Network’s year 1 findings on bacterial meningitis in Jordan, Egypt, and Iraq (2023-2024)

**DOI:** 10.1016/j.ijregi.2026.100896

**Published:** 2026-04-16

**Authors:** Lamia Al-Kershi, Deema Al Bakri, Zeina Abdel Majeed, Sara Abu Khudair, Magid Al Gunaid, Dominique A. Caugant, Muhamed-Kheir Taha, Saber Yezli, Tarek Al-Sanouri, Maya Joukhadar, Rund Al-Qaseer, Ener Cagri Dinleyici, Areej Shoubaki, Marwa Abdel Shafy, Sahar Sami, Rawya Mohamed, Hussein Al Shammary, Ahmed Abdulsattar Salman, Florence Coste, Alp Dogu, Laure Badet, Amine Amiche, Ray Borrow

**Affiliations:** 1 Eastern Mediterranean Public Health Network, Amman, Jordan; 2 Division for Infection Control, Norwegian Institute of Public Health, Oslo, Norway; 3 Institut Pasteur, Invasive bacterial infections unit, Paris, France; 4 Biostatistics, Epidemiology, and Scientific Computing Department, King Faisal Specialist Hospital and Research Centre, Riyadh, Saudi Arabia; 5 Eskisehir Osmangazi University Faculty of Medicine, Department of Pediatrics, Eskisehir, Türkiye; 6 Surveillance Department, Ministry of Health, Amman, Jordan; 7 Acute Infections Neurological Disease Control Program, Ministry of Health, Cairo, Egypt; 8 Ministry of Health, Cairo, Egypt; 9 Central Public Health Laboratories, Ministry of Health, Baghdad, Iraq; 10 Communicable Diseases Control Center, Ministry of Health, Baghdad, Iraq; 11 Sanofi Vaccines, Global Medical, Lyon, France; 12 Sanofi Vaccines, Global Medical, Dubai, UAE; 13 Meningococcal Reference Unit, UK Health Security Agency, Manchester Royal Infirmary, Manchester, UK

**Keywords:** Bacterial meningitis, Surveillance, Eastern Mediterranean Region, Invasive bacterial diseases, Middle East and North Africa, Real-time PCR

## Abstract

**Objectives:**

Vaccine-preventable invasive bacterial diseases remain a public health concern in the Middle East and North Africa (MENA) region. To address surveillance and diagnostic gaps, the Meningitis and Septicemia Mapping Network (MenMap) was launched as a prospective, multicenter sentinel surveillance system to strengthen laboratory capacity and regional collaboration in Jordan, Egypt, and Iraq. This study reports the first-year MenMap findings (December 2023 to November 2024), describing bacterial meningitis epidemiology, pathogen distribution, vaccination status, clinical presentation, and outcomes among laboratory-confirmed cases in Jordan, Egypt, and Iraq.

**Methods:**

Prospective sentinel hospital-based surveillance was implemented in 16 hospitals, targeting children aged 1 month to 18 years. Suspected cases were defined according to World Health Organization criteria and included patients presenting with clinical signs of meningitis and undergoing cerebrospinal fluid and/or blood sampling. Standardized protocols included clinical evaluations, real-time polymerase chain reaction for pathogen detection, and continuous monitoring systems. An external quality assessment scheme ensured laboratory diagnostic quality and accuracy.

**Results:**

Between December 2023 and November 2024, 2104 suspected cases were reported, with 191 (9%) confirmed*. S. pneumoniae* was the most prevalent (90.0%), followed by *H. influenzae* (7.9%) and *N. meningitidis* (2.1%). Most confirmed cases were from Iraq (63.3%), and 62% across the three countries occurred in children aged <5 years.

**Conclusion:**

MenMap strengthens bacterial meningitis surveillance in the MENA region and provides critical evidence to support vaccine policy decisions, including expansion of pneumococcal and *Haemophilus influenzae* type b immunization programs, alongside improvements in pathogen detection and characterization.

## Highlights

• Multi-country meningitis surveillance in Jordan, Egypt, and Iraq.
• Streptococcus pneumoniae accounted for 90% of confirmed cases.
• Most cases occurred in children aged under 5 years.
• Polymerase chain reaction–based diagnostics supported pathogen detection.
• Findings inform regional action toward World Health Organization’s Defeating Meningitis 2030.

## Introduction

Bacterial meningitis remains a major global health concern, especially among infants and children, leading to high mortality and severe long-term sequelae such as hearing loss and cognitive impairment [1]. Although the highest burden occurs in the “meningitis belt” of sub-Saharan Africa [2], the Middle East and North Africa (MENA) region also experiences endemic and epidemic occurrences of invasive bacterial diseases (IBDs), primarily caused by *Neisseria meningitidis, Streptococcus pneumoniae*, and *Haemophilus influenzae* type b (Hib) [3].

In many Eastern Mediterranean Region (EMR) countries, surveillance remains fragmented, with inconsistent case definitions and limited laboratory capacity, particularly, for meningococcal disease [4]. Restricted access to molecular diagnostics such as real-time polymerase chain reaction (PCR) hampers timely pathogen confirmation, resulting in underestimation of disease burden and inadequate outbreak response [1].

In this context, bacterial meningitis continues to impose a substantial global burden. It was estimated to cause 236,000 deaths in 2019, including 112,000 among children under 5 years of age [1]. The World Health Organization (WHO) Defeating Meningitis by 2030 roadmap outlines five key pillars: prevention and epidemic control, diagnosis and treatment, surveillance, care for those affected, and advocacy and engagement [5]. However, surveillance capacity in the EMR remains limited, with persistent laboratory and reporting gaps and substantial variation in pneumococcal conjugate vaccine (PCV) uptake across countries. For example, although some countries have incorporated PCV into routine immunization schedules, others have yet to introduce it, contributing to uneven protection across the region [6].

The Meningitis and Septicemia Mapping Network (MenMap), initiated by the Global Health Development (GHD) in collaboration with Sanofi and National Ministries of Health, aims to address these gaps by contributing to the pillars of the WHO roadmap through strengthening surveillance, enhancing laboratory diagnostics, building regional capacity, and establishing a multi-country platform for data sharing and research [7].

### Study objectives

The paper presents the first-year findings of MenMap surveillance (December 2023 to November 2024), describing the epidemiology and molecular characteristics of *N. meningitidis, S. pneumoniae*, and *H. influenzae* in Jordan, Egypt, and Iraq. Specific objectives were to (i) strengthen collaboration among participating countries, (ii) enhance diagnostic capacity through real-time PCR, (iii) integrate laboratory and epidemiologic data to improve quality and utility, and (iv) facilitate generation of regional data to support national and international decision-making.

## Methods

### Study design and setting

A prospective, hospital-based sentinel surveillance design was implemented across Jordan, Egypt, and Iraq from December 2023 to November 2024. Sentinel hospitals were identified with national stakeholders based on diagnostic capacity, geographic distribution, and referral coverage to generate standardized data on vaccine-preventable IBD caused by *N. meningitidis, S. pneumoniae*, and Hib. A total of 16 participating hospitals (four in Jordan, four in Egypt, and eight in Iraq) functioned as secondary or tertiary referral centers, equipped to collect, store, and transport cerebrospinal fluid (CSF) and blood specimens for molecular testing at the respective Central Public Health Laboratories (CPHLs) under the MenMap surveillance framework. The hospitals’ details are provided in Table 1.

**Table 1:** Distribution of polymerase chain reaction–confirmed bacterial meningitis cases by demographic characteristics and sentinel site contribution (December 2023 to November 2024).

Factor	Country							
	Regional		Jordan		Egypt		Iraq	
	N	%	n	%	n	%	n	%
**Gender**								
Female	81	42.4	13	48.1	15	34.9	53	43.8
Male	110	57.6	14	51.9	28	65.1	68	56.2
**Age group**								
<1 year	56	29.3	18	66.7	13	30.2	25	20.7
1-5 years	62	32.5	7	25.9	3	7.0	52	43.0
6-14 years	65	34.0	2	7.4	19	44.2	44	36.3
15-18 years	8	4.2	0	0.0	8	18.6	0	0.0

Sentinel sites^a^			
Country	Hospital name	N	%
**Jordan**	Al Basheer Hospital	11	40.7
	Al Karak Hospital	3	11.1
	Zarqa Hospital	4	14.8
	Princess Rahma Pediatric Hospital	9	33.3
**Egypt**	Abbasia Fever Hospital	33	76.8
	Hurghada Fever Hospital	0	0.0
	Sohag Fever Hospital	5	11.6
	Zagazig Fever Hospital	5	11.6
**Iraq**	Al Kadhimiya Pediatric Hospital	5	4.1
	Al-Zahraa Pediatrics Teaching Hospital	9	7.4
	Basra Pediatric hospital	2	1.7
	Central Pediatric Hospital	53	43.8
	Elwia Pediatric Hospital	4	3.3
	Ibn Al-Balady Pediatric Hospital	12	9.9
	Karbala Pediatric Hospital	35	28.9
	Kirkuk Pediatric Hospital	1	0.8

a Percentages reflect each hospital’s contribution to confirmed cases within the same country.

### Target population and case definition

Children aged 1 month to 18 years presenting with suspected bacterial meningitis were eligible for inclusion in the population at highest risk of invasive bacterial meningitis. A suspected case was defined according to WHO guidelines [8] as a sudden onset of fever (≥38.0°C rectal or ≥37.5°C axillary) plus one or more of the following: neck stiffness, altered consciousness, bulging fontanelle (infants), new-onset seizures, severe headache, photophobia, or vomiting. On the other hand, a confirmed case was defined as a suspected case with laboratory confirmation of *N. meningitidis, S. pneumoniae*, or *H. influenzae* by real-time PCR and/or culture from CSF or blood specimens.

**Inclusion criteria:** Meeting the clinical definition, having CSF/blood collected, residing within the catchment area, and provided informed consent.
**Exclusion criteria:** Failure to meet the clinical definition, insufficient sample volume, attribution of symptoms solely to preexisting neurologic conditions, or lacked consent.

Although MenMap includes septicemia within its broader scope, this analysis focused on clinically suspected meningitis cases. Cases presenting solely with septicemia without meningitis were not included in this study.

### Data collection

Clinical and epidemiologic data were recorded at presentation using a standardized MenMap care report form (CRF), capturing demographics, symptoms, vaccination, and antibiotic exposure. CSF was obtained via lumbar puncture under aseptic conditions; when not feasible due to clinical or social constraints, blood samples were collected to support the diagnosis of invasive bacterial infection. Samples were labeled with unique IDs and transported under cold chain (2-8°C) to CPHL for PCR and, when possible, culture.

### Standardized surveillance protocol

All sites followed a unified MenMap surveillance protocol jointly with the Ministries of Health and the MenMap Steering and Scientific Committees. The protocol standardized case identification, specimen handling, laboratory workflows, and reporting mechanisms. Clinical and laboratory staff received pre-implementation training, supported by job aids and supervisory visits.

### Laboratory procedures and analytic phases

All laboratories followed the MenMap protocol aligned with WHO recommendations. Real-time PCR served as the primary diagnostic tool targeting conserved genes: *ctrA* and *sodC* for *N. meningitidis, lytA* for *S. pneumoniae*, and *hpd* for *H. influenzae*). Results were interpreted as positive (cycle threshold [Ct] ≤35), indeterminate (36-40; retested), or negative (>40). When feasible, CSF and blood were cultured by standard bacteriological methods; isolates were preserved at −80°C for subtyping. Laboratory workflows maintained unidirectional movement from extraction to amplification to minimize contamination. Samples were logged, validated for quality, processed in nuclease-free conditions, and amplified using validated PCR kits with control verification. Results were electronically reviewed against reference Ct thresholds.

### External quality assessment (EQA) process

EQA scheme was coordinated with the Institut Pasteur to evaluate inter-laboratory performance, reproducibility of Ct values, and protocol adherence. Discrepancies were addressed through corrective actions, refresher training, and formal feedback.

### Biosafety considerations

All laboratory activities adhered to biosafety level 2 precautions, using personal protective equipment, certified class II biosafety cabinet, and appropriate waste disposal. Staff were encouraged to maintain immunization against invasive bacterial pathogens.

### Data management

Data management followed a unified protocol ensuring cross-country comparability. CRFs captured demographic, clinical, and laboratory variables and were entered into secure national databases linked to patients’ IDs. Data entry staff, clinicians, and laboratorians received training on standardized recording, data security, and reporting timelines. Data ownership remained under national authority control, with anonymized data sets centrally managed by GHD for analysis.

### Statistical analysis

Descriptive statistics were performed using the SPSS version 29. Continuous variables are summarized using appropriate measures of central tendency, and categorical variables are presented as frequencies and percentages. Figures and tables were generated in Microsoft Excel, and Power BI dashboards supported data visualization and routine monitoring. Supplementary tables and figures are designated with the prefix “S” (e.g. S1, Figure S1) and are provided in the Supplementary Material.

## Results

**Figure 1:**
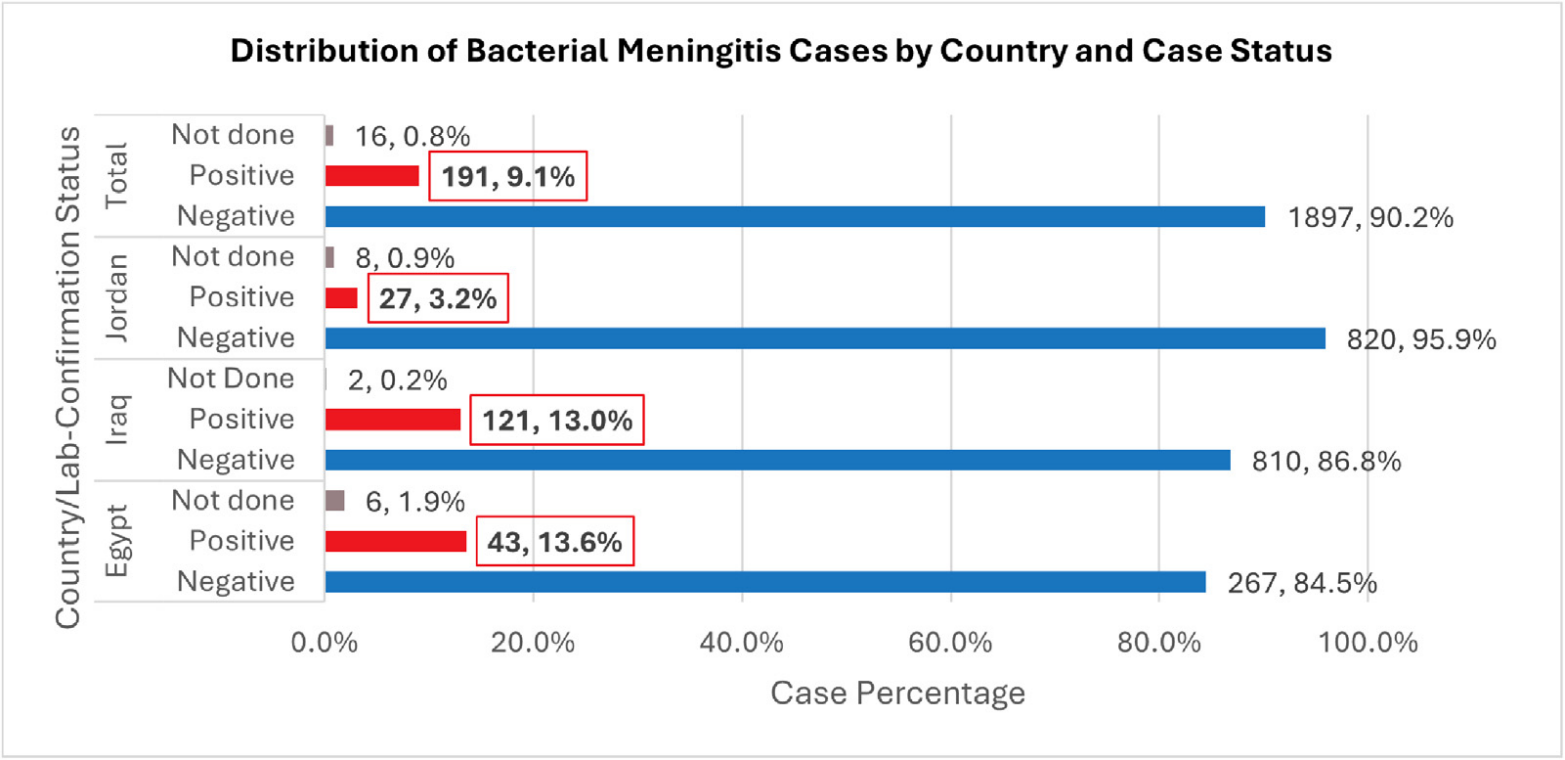
Distribution of bacterial meningitis cases by country and case status (December 2023 to November 2024).

Between December 2023 and November 2024, a total of 2104 suspected meningitis cases were reported across 16 sentinel hospitals in Jordan, Egypt, and Iraq. Of these, 191 (9.1%) were PCR-confirmed. As shown in Figure 1, Egypt recorded the highest confirmation rate (13.6%), followed by Iraq (13.0%) and Jordan (3.2%).

### Pathogen distribution

**Figure 2:**
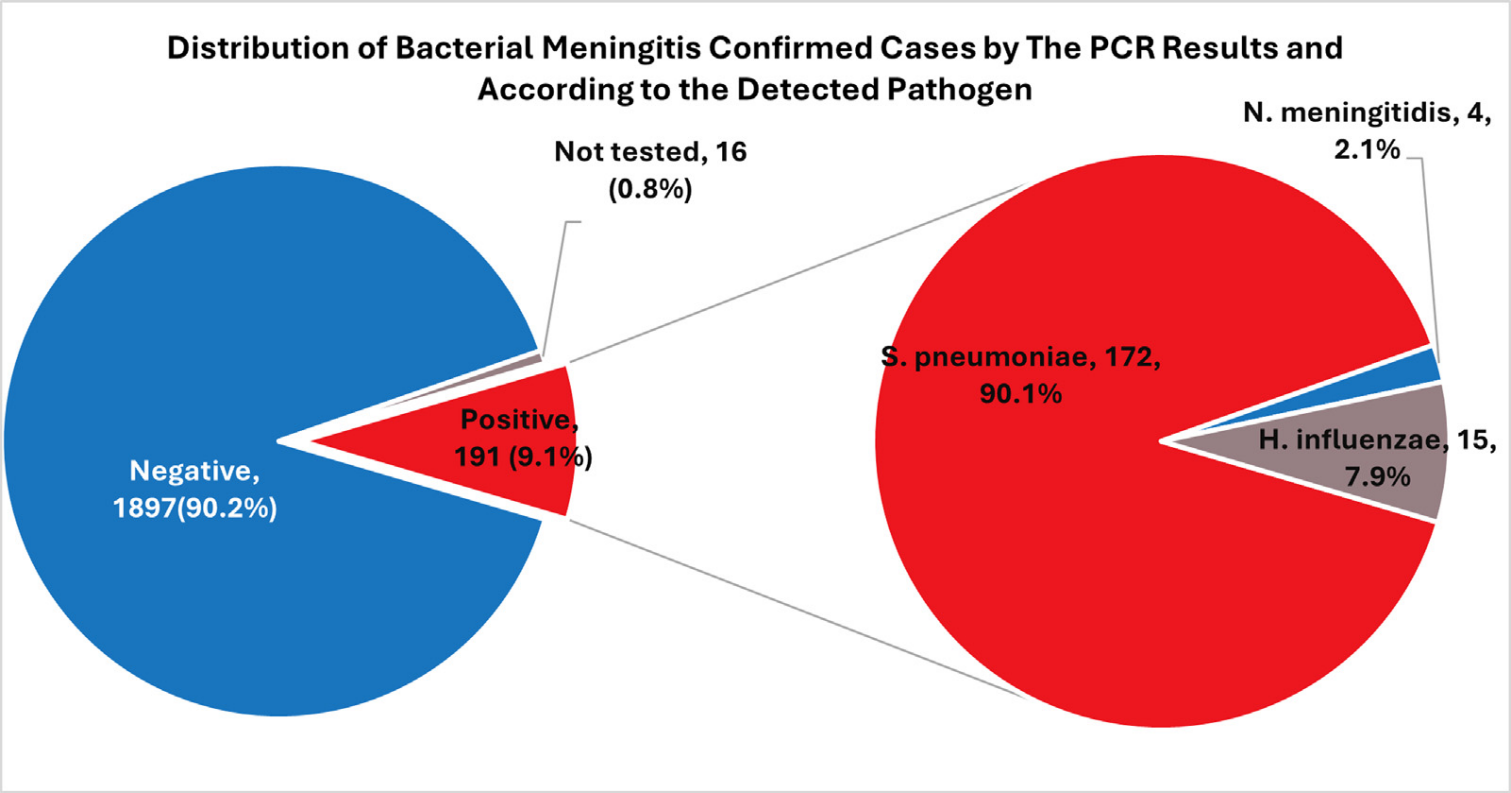
Distribution of confirmed bacterial meningitis cases by PCR results and detected pathogen (December 2023 to November 2024). PCR, polymerase chain reaction.

Among confirmed cases, *S. pneumoniae* predominated with 172 (90.1%) cases, followed by *H. influenzae* with 15 (7.9%) cases, and *N. meningitidis* with four (2.1%) cases (Figure 2). The pattern was consistent across countries: *S. pneumoniae* accounted for 92.6% in Jordan, 90.7% in Egypt, and 89.3% in Iraq, whereas *H. influenzae* contributed 7-10% of cases. *N. meningitidis* was detected only in Egypt and Iraq (Table S1). The number of *N. meningitidis* cases was low (n = 4), limiting detailed epidemiological interpretation.

Serogrouping identified two *N. meningitidis* serogroup B cases (Egypt), one serogroup A (Iraq), and one undetermined case (Egypt) (Figure S1). CSF remained the primary diagnostic specimen (91.1% of confirmed cases), with blood accounting for 8.9% (Table S2).

### Demographics

As shown in Table 1, males accounted for a slightly higher proportion of confirmed cases (57.6%) than females (42.4%). Age distribution varied by country: Jordan was dominated by infants aged <1 year (66.7%), Egypt by children aged 6-14 years (44.2%), and Iraq by children aged 1-5 years (43.0%).

In Jordan, most confirmed cases were reported from Al-Bashir Hospital (40.7%) and Princess Rahma Pediatric Hospital (33.3%). In Egypt, Abbassia Fever Hospital contributed the majority of confirmed cases (76.8%), whereas Zagazig and Sohag Fever Hospitals accounted for 23.2% of confirmed cases.

### Seasonality and time trend

**Figure 3:**
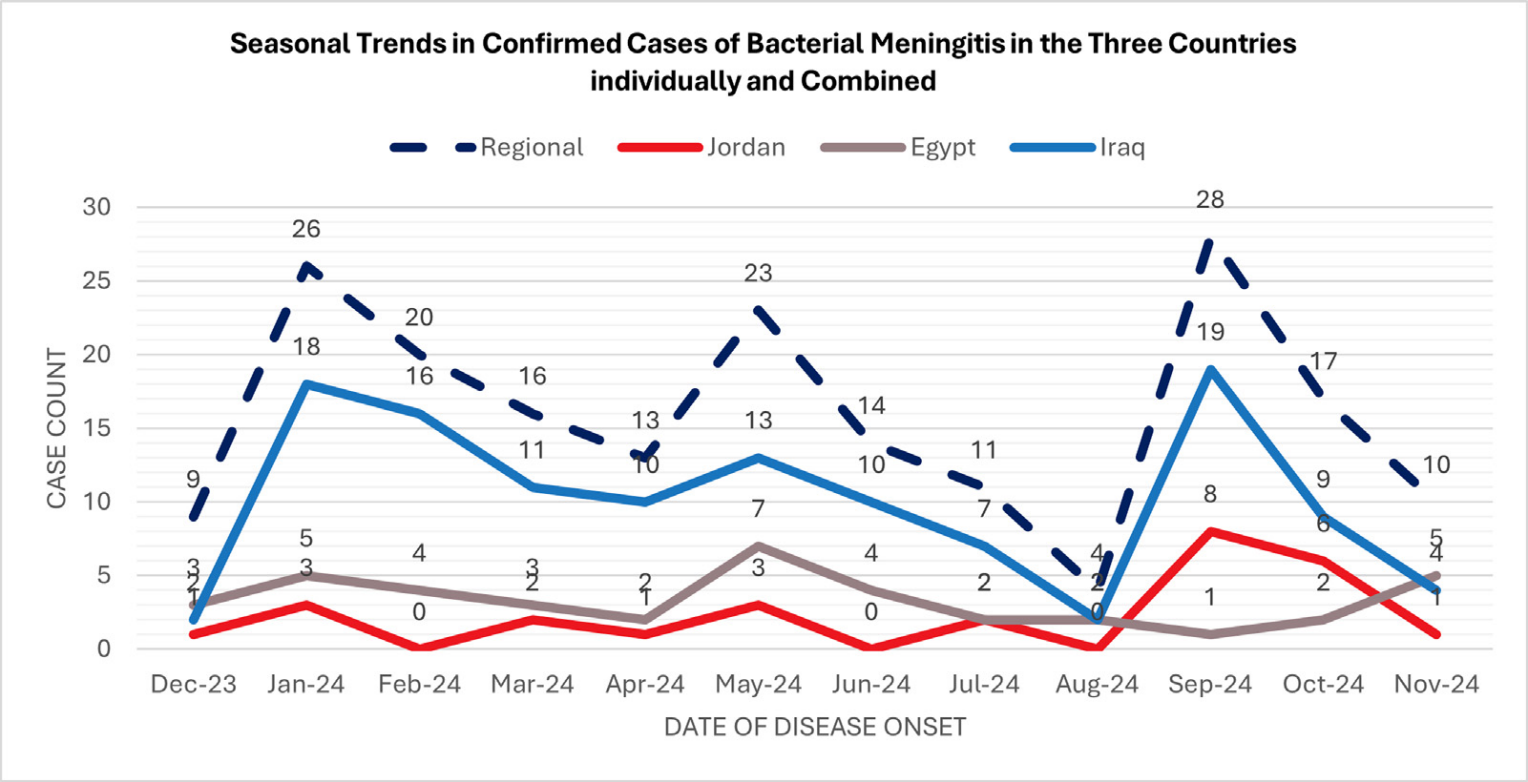
Seasonal trends in confirmed bacterial meningitis cases in the three countries individually and combined (late 2023 to November 2024).

Seasonal variation in confirmed bacterial meningitis cases was assessed using reported symptom onset dates (Figure 3). Regional trends showed two peaks: January 2024 and September 2024. Country-specific variations in temporal patterns were observed across Jordan, Egypt, and Iraq.

### Vaccination status

Vaccination history was obtained through caregiver reports and vaccination cards where available. Vaccination data should be interpreted with caution due to a high proportion of missing or unknown records, with over 40% of entries marked as “unknown” across all countries (Tables S3-S5).

In Jordan, approximately one-third of suspected and confirmed cases had documented Hib vaccination, whereas no meningococcal or pneumococcal vaccination was reported, reflecting the national schedule. In Egypt, 13% of suspected cases reported a history of meningococcal vaccination, mainly, bivalent polysaccharide MenA + C vaccine administered through school-entry campaigns, whereas Hib vaccination was documented in about one-third of cases. In Iraq, 21% reported bacterial meningitis-related vaccination, including MenACWY conjugate among meningococcal recipients, whereas pneumococcal (1.7%) and Hib vaccination (18.3%) were infrequently reported. Over half (55.9%) of cases had no vaccination record, and 68.6% of PCR-confirmed cases lacked documentation, reflecting limitations in data completeness.

### Clinical symptoms

PCR-confirmed cases presented with classical bacterial meningitis symptoms (Table S6). Fever was the most common symptom (94.2%), followed by vomiting (50.8%), headache (40.8%), neck stiffness (39.3%), and seizures (34.6%). Country-specific variations were observed. In Jordan, vomiting (40.7%) and lethargy (33.3%) were most frequent. In Egypt, headache (62.8%) and vomiting (55.8%) predominated, whereas in Iraq, vomiting (51.2%) and neck stiffness (45.5%) were most common. Less frequent but clinically relevant findings included photophobia, bulging fontanelle, and purpuric or hemorrhagic lesions.

### Antibiotic administration

Antibiotic therapy was a central component of case management (Table S7), with combination regimens frequently used. Ceftriaxone was administered in over 90% of confirmed cases regionally and universally in Egypt. Vancomycin use varied, highest in Iraq (75.2%) compared with Jordan (26.4%) and Egypt (12.0%). Amoxicillin was rarely prescribed (<4%), whereas penicillin G and oxacillin were not reported. Other antibiotics, including cefotaxime, ampicillin, and meropenem, were used infrequently.

### Confirmed cases outcomes

Outcomes among PCR-confirmed cases are summarized in Table 2. Categories included the following: on treatment, recovered, referred, deceased, and unknown (patients discharged or lost to follow-up).

**Table 2:** Patient outcomes among confirmed bacterial meningitis cases in Jordan, Egypt, and Iraq (December 2023 to November 2024).

Country	Patient outcome									
	On treatment		Recovered		Referred		Dead		Unknown	
	n	%	n	%	N	%	n	%	N	%
**Jordan**	24	88.9	3	11.1	0	0.0	0	0.0	0	0.0
**Egypt**	5	11.6	28	65.2	5	11.6	4	9.3	1	2.3
**Iraq**	67	55.4	2	1.7	52	42.9	0	0.0	0	0.0

In Jordan, 88.9% remained on treatment and 11.1% recovered, with no deaths or referrals reported. In Egypt, 65.2% recovered, whereas 11.6% were on treatment and 11.6% were referred. Four deaths (9.3%) were recorded, and 2.3% were categorized as unknown. In Iraq, 55.4% remained on treatment, 42.9% were referred, and 1.7% recovered at the time of reporting.

### EQA results

The non-culture EQA panel comprised seven lyophilized samples: *N. meningitidis* (non-groupable and serogroups B, C, W, and X), *S. pneumoniae*, and *H. influenzae* type b. All laboratories correctly identified *S. pneumoniae* and *N. meningitidis* serogroup B. *H. influenzae* was accurately detected at the species level by all laboratories, although only 75% correctly identified it as type b. Performance was lowest for non-groupable *N. meningitidis* (25% accuracy), likely due to the absence of the ctrA gene in some strains. Overall, species-level accuracy was 86%, and genogroup-level accuracy was 80%.

The 2024 EQA results confirmed reliable PCR implementation and diagnostic capacity while highlighting the need for harmonized protocols, standardized reagent panels, and continued training across laboratories.

## Discussion

This study found that *S. pneumoniae* remains the leading cause of bacterial meningitis across Jordan, Egypt, and Iraq, consistent with global evidence showing pneumococcus as the primary pathogen driving IBD [9]. The lower contribution of *H. influenzae* likely reflects the impact of Hib vaccination programs, although residual cases suggest gaps in coverage or access. The number of *N. meningitidis* cases was limited (n = 4), restricting detailed epidemiologic interpretation. However, its detection across multiple serogroups underscores the importance of continued surveillance.

Disease predominance among young children is consistent with established vulnerability in early life, highlighting the importance of pediatric prevention strategies. However, interpretation of vaccination-related findings is limited by substantial gaps in documentation, with a high proportion of records classified as “unknown.” As such, conclusions regarding vaccination coverage or effectiveness should be interpreted with caution. Although variations in national immunization programs may contribute to observed differences, the current data do not allow robust analytical assessment of vaccine impact.

Serogroup characterization of *N. meningitidis* provided limited but relevant information for surveillance, although the small number of cases precludes broader conclusions. Pneumococcal serotyping was not performed, limiting assessment of circulating serotypes and their potential alignment with vaccine coverage.

Worldwide, bacterial meningitis incidence and mortality have declined since 1990, largely due to the introduction of conjugate vaccines, improved health care access, and strengthened surveillance systems [10]. Nevertheless, meningitis continues to impose a significant burden, particularly, in low- and middle-income settings where diagnostic capacity and timely access to care remain limited [11]. Although Hib meningitis has been substantially reduced in high-income countries [12], pneumococcal meningitis remains a leading vaccine-preventable mortality in under-immunized populations [13].

In the MENA region, epidemiologic data remain fragmented [14], and available data confirm *S. pneumoniae* as the dominant pathogen, consistent with the MenMap findings [15]. A regional systemic review highlighted uneven vaccination impact [16]. Studies from Saudi Arabia and Lebanon reported similar patterns, with pneumococcus leading and Hib persisting where coverage is incomplete [17,18]. Meningococcal disease, although relatively infrequent, remains a recognized risk in specific contexts such as mass gatherings [18,19].

Compared with the sub-Saharan African “meningitis belt,” where *N. meningitidis* has historically caused large-scale epidemics, the EMR exhibits a lower overall burden [20]. After the introduction of MenAfriVac, serogroup A disease declined substantially, although other serogroups continue to emerge in outbreak settings [21]. In contrast, the limited number of meningococcal cases observed in this study aligns with the lower endemicity in the region while still supporting the need for ongoing monitoring [22].

The observed predominance of pneumococcal disease, alongside the persistence of Hib, highlights the importance of strengthening vaccination strategies in the region. However, given the limitations in vaccination data completeness, these findings should be interpreted cautiously and not as a direct measure of vaccine performance. Expanding access to PCVs and sustaining Hib immunization remain important public health priorities.

EQA results demonstrate generally good laboratory performance in pathogen detection; however, variability in genogroup identification and challenges in detecting non-groupable *N. meningitidis* indicate the need for further standardization, refinement of molecular targets, and continued capacity building across laboratories.

### Context of National vaccination programs

The vaccination context was reviewed using the WHO/UNICEF Joint Reporting Process [23]. However, data on Hib or pneumococcal conjugate vaccination programs were obtained directly from national immunization schedules and verified with the respective Ministries of Health.

**Jordan:** The Hib vaccine is part of the national immunization program (coverage ∼95%), but neither PCV nor meningococcal vaccine are routinely administered.

**Egypt:** Meningococcal vaccination has been mandatory for schoolchildren since 1982 using bivalent (A + C) polysaccharide vaccines. The quadrivalent ACWY conjugate vaccine was introduction in 2002 for Hajj and Umrah pilgrims. The Hib vaccine was added to the routine schedule in 2014 (at 2, 4, and 6 months), although PCV remains outside the national program.

**Iraq:** The pentavalent vaccine (including Hib) and PCV13 are part of the routine schedule (2, 4, and 6 months). The ACWY vaccine is not part of the national program but is used selectively for high-risk groups and during mass gatherings.

### Limitations

Several limitations should be considered when interpreting these findings. Data gaps were noted, particularly, in vaccination history, where many records were marked “unknown” due to reliance on caregiver recall or incomplete documentation, limiting meaningful assessment of vaccination coverage or effectiveness. Loss to follow-up among early discharges or transfers reduced completeness of outcome data, and neurologic sequelae were not assessed, limiting evaluation of long-term disease burden.

Challenges in CSF collection, especially among infants where lumbar puncture was contraindicated or declined due to social beliefs regarding potential complications, including paralysis, likely reducing diagnostic yield. In such cases, blood samples were used to support diagnosis. The relatively low laboratory confirmation rate may also reflect pre-admission antibiotic use, delayed presentation, and variability in specimen quality. Although PCR improved detection, these factors introduce uncertainty.

Pneumococcal serotyping was not performed, limiting assessment of circulating serotypes and their potential coverage by available vaccines. Laboratories showed reduced performance in identifying non-groupable *N. meningitidis* during EQA, highlighting the need to refine molecular targets. Operational constraints such as delayed or incomplete data entry may also have contributed to occasional misclassification. Finally, sentinel sites, although geographically diverse, do not represent full national populations, limiting generalizability.

## Conclusion and Recommendations

This multi-country surveillance confirmed *S. pneumoniae* as the predominant cause of bacterial meningitis across Jordan, Egypt, and Iraq, accounting for nearly 90% of confirmed cases, whereas *H. influenzae* type b and *N. meningitidis* contributed smaller proportions. Most cases occurred in children under 5 years of age. The study underscored the values of real-time PCR in improving pathogen detection and serogroup identification despite previous antibiotic exposure. Strengthening meningitis surveillance in the EMR through timely case detection, standardized laboratory confirmation, and data integration remains a regional priority.

Sustained progress requires investments in PCR diagnostics, maintaining robust EQA, and improving specimen collection and vaccination documentation. Broader pneumococcal and Hib vaccine coverage and meningococcal preparedness in high-risk settings are essential. Integrating MenMap data into national antimicrobial-resistance monitoring systems would further support evidence-based treatment policies and guide antibiotic stewardship, complementing efforts to achieve the WHO Defeat Meningitis by 2030 goals.

## Declaration of competing interest

Magid Al-Gunaid, Rund Al-Qaseer, Zeina AbdelMajeed, Deema Bakri, Sara Abu Khdair, and Lamia Al-Kershi are employed by GHD|EMPHNET, an organization that conducts epidemiological studies on infectious and non-infectious diseases, including those funded by Sanofi and other organizations, outside the scope of this work. Tarek Al-Sanouri and Maya Joukhadar were employees of GHD|EMPHNET during the course of this project year. Muhamed-Kheir Taha performs contract work for the Institut Pasteur funded by GSK, Pfizer, and Sanofi and holds a patent (NZ630133A) with GSK titled “Vaccines for Serogroup X Meningococcus.” Ray Borrow conducts contract research on behalf of UKHSA for GSK, PATH, Pfizer, and Sanofi. Ener Cagri Dinleyici performs contact work for Eskisehir Osmangazi University funded by Glaxo Smith Kline, Pfizer, Merck Sharp&Dohme, and Sanofi. Dominique A. Caugant and Saber Yezli have no conflicts of interest related to this project. Amine Amiche, Florence Coste, Alp G. Dogu, and Laure Badet are employees of Sanofi.

## Appendix Supplementary materials

Image, application 1

Image, application 2

Image, application 3

Image, application 4

Image, application 5

Image, application 6

Image, application 7

Image, application 8

## Footnotes

Supplementary material associated with this article can be found, in the online version, at doi:10.1016/j.ijregi.2026.100896.
