# Supplementary material for: Strengthening regional surveillance: MenMap Network’s year 1 findings on bacterial meningitis in Jordan, Egypt, and Iraq (2023-2024)
Source: IJID Reg. 2026 Apr 16;19:100896. doi: 10.1016/j.ijregi.2026.100896 (PMC13147366; doi:10.1016/j.ijregi.2026.100896)
Supplement: Supplementary file 1 [file mmc1.docx]

Tables and Figures Captions (Supplementary)

**Figure S1**: Distribution of Bacterial Meningitis Confirmed Cases by the Detected Pathogen (December 2023–November 2024)

**Table S1:** Distribution of The Detected Pathogens Among Bacterial Meningitis Cases in Jordan, Egypt, and Iraq (December 2023–November 2024).

**Table S2**: Distribution of PCR-Confirmed Bacterial Meningitis Cases by Type of Specimen in the Three Countries (December 2023–November 2024).

**Table S3:** Vaccination Status and Type in Bacterial Meningitis Cases in Jordan (December 2023–November 2024)

**Table S4:** Vaccination Status and Type in Bacterial Meningitis Cases in Egypt (December 2023–November 2024)

**Table S5:** Vaccination Status and Type in Bacterial Meningitis Cases in Iraq (December 2023–November 2024)

**Table S6:** Distribution of Presenting Signs and Symptoms Among PCR-Confirmed Bacterial Meningitis Cases in Jordan, Egypt, and Iraq (December 2023–November 2024)

**Table S7:** Distribution of antibiotics administered among PCR-confirmed bacterial meningitis cases in Jordan, Egypt, and Iraq (December 2023-November 2024)
