# Supplementary material for: Strengthening regional surveillance: MenMap Network’s year 1 findings on bacterial meningitis in Jordan, Egypt, and Iraq (2023-2024)
Source: IJID Reg. 2026 Apr 16;19:100896. doi: 10.1016/j.ijregi.2026.100896 (PMC13147366; doi:10.1016/j.ijregi.2026.100896)
Supplement: Supplementary file 2 [file mmc2.docx]

| **Country** | **Detected Pathogen** | | | | | | | |
| --- | --- | --- | --- | --- | --- | --- | --- | --- |
|  | ***S. pneumoniae*** | | ***H. influenzae*** | | ***N. meningitidis*** | | **Total** | |
|  | **n** | **%** | **n** | **%** | **n** | **%** | **N** | **%** |
| **Jordan** | 25 | 92.6 | 2 | 7.4 | 0 | 0.0 | 27 | 3.2 |
| **Egypt** | 39 | 90.7 | 1 | 2.3 | 3 | 7.0 | 43 | 13.6 |
| **Iraq** | 108 | 89.3 | 12 | 9.9 | 1 | 0.8 | 121 | 13.0 |
