# Supplementary material for: Strengthening regional surveillance: MenMap Network’s year 1 findings on bacterial meningitis in Jordan, Egypt, and Iraq (2023-2024)
Source: IJID Reg. 2026 Apr 16;19:100896. doi: 10.1016/j.ijregi.2026.100896 (PMC13147366; doi:10.1016/j.ijregi.2026.100896)
Supplement: Supplementary file 3 [file mmc3.docx]

| **Specimen Type** | **Country** | | | | | | | |
| --- | --- | --- | --- | --- | --- | --- | --- | --- |
|  | **Regional** | | **Jordan** | | **Egypt** | | **Iraq** | |
|  | **N** | **%** | **n** | **%** | **n** | **%** | **n** | **%** |
| Cerebrospinal Fluid (CSF) | 174 | 91.1 | 25 | 92.6 | 43 | 100.0 | 106 | 87.6 |
| Blood | 17 | 8.9 | 2 | 7.4 | 0 | 0.0 | 15 | 12.4 |
