# Supplementary material for: Strengthening regional surveillance: MenMap Network’s year 1 findings on bacterial meningitis in Jordan, Egypt, and Iraq (2023-2024)
Source: IJID Reg. 2026 Apr 16;19:100896. doi: 10.1016/j.ijregi.2026.100896 (PMC13147366; doi:10.1016/j.ijregi.2026.100896)
Supplement: Supplementary file 4 [file mmc4.docx]

| Vaccination Status | PCR Result | | | | | | | |
| --- | --- | --- | --- | --- | --- | --- | --- | --- |
|  | Negative | | Positive | | Not Done | | Total | |
|  | n | % | N | % | n | % | N | % |
| Ever Been Vaccinated to Bacterial Meningitis | 292 | 53.5 | 9 | 33.3 | 1 | 12.5 | 302 | 35.3 |
| **Meningococcal Vaccine** | | | | | | | | |
| No | 546 | 66.6 | 16 | 59.3 | 4 | 50.0 | 566 | 66.2 |
| Unknown ** | 274 | 33.4 | 11 | 40.7 | 4 | 50.0 | 289 | 33.8 |
| **Pneumococcal Conjugate Vaccine Type** | | | | | | | | |
| No | 548 | 66.8 | 16 | 59.3 | 4 | 50.0 | 568 | 66.4 |
| Unknown | 272 | 33.2 | 11 | 40.7 | 4 | 50.0 | 287 | 33.6 |
| ***Haemophilus Influenzae* Type b** | | | | | | | | |
| Yes | 292 | 35.6 | 9 | 33.3 | 1 | 12.5 | 302 | 35.3 |
| No | 257 | 31.3 | 6 | 22.2 | 3 | 37.5 | 266 | 31.1 |
| Unknown ** | 271 | 33.1 | 12 | 44.5 | 4 | 50.0 | 287 | 33.6 |
| ** Unknown cases are derived from the self-reported information provided by the patients or their guardians. | | | | | | | | |
