# Supplementary material for: Strengthening regional surveillance: MenMap Network’s year 1 findings on bacterial meningitis in Jordan, Egypt, and Iraq (2023-2024)
Source: IJID Reg. 2026 Apr 16;19:100896. doi: 10.1016/j.ijregi.2026.100896 (PMC13147366; doi:10.1016/j.ijregi.2026.100896)
Supplement: Supplementary file 5 [file mmc5.docx]

| Vaccination Status | PCR Result | | | | | | | |
| --- | --- | --- | --- | --- | --- | --- | --- | --- |
|  | Negative | | Positive | | Not Done | | Total | |
|  | n | % | N | % | n | % | N | % |
| Ever Been Vaccinated to Bacterial Meningitis | 113 | 42.3 | 21 | 48.8 | 2 | 33.3 | 136 | 43.0 |
| **Meningococcal Vaccine** | | | | | | | | |
| Yes* | 36 | 13.5 | 6 | 14.0 | 0 | 0.0 | 42 | 13.3 |
| *Conjugate* | *7* | *19.4* | *0* | *0.0* | *0* | *0.0* | *7* | *16.7* |
| *Polysaccharide (MenA+C)* | *29* | *80.6* | *6* | *100.0* | *0* | *0.0* | *35* | *83.3* |
| No | 125 | 46.8 | 22 | 51.2 | 4 | 66.7 | 151 | 47.8 |
| Unknown ** | 106 | 39.7 | 15 | 34.9 | 2 | 33.3 | 123 | 38.9 |
| **Pneumococcal Conjugate Vaccine Type** | | | | | | | | |
| No | 171 | 64.0 | 32 | 74.4 | 5 | 83.3 | 208 | 65.8 |
| Unknown | 96 | 36.0 | 11 | 25.6 | 1 | 16.7 | 108 | 34.2 |
| **Haemophilus Influenzae Type b** | | | | | | | | |
| Yes | 89 | 33.3 | 18 | 41.9 | 2 | 33.3 | 109 | 34.5 |
| No | 79 | 29.6 | 14 | 32.6 | 2 | 33.3 | 95 | 30.1 |
| Unknown ** | 99 | 37.1 | 11 | 25.6 | 2 | 33.3 | 112 | 35.4 |
| *Rates are based on having the denominator (total) equal to those who received the vaccine type.  ** Unknown cases are derived from the self-reported information provided by the patients or their guardians. | | | | | | | | |
