# Supplementary material for: Strengthening regional surveillance: MenMap Network’s year 1 findings on bacterial meningitis in Jordan, Egypt, and Iraq (2023-2024)
Source: IJID Reg. 2026 Apr 16;19:100896. doi: 10.1016/j.ijregi.2026.100896 (PMC13147366; doi:10.1016/j.ijregi.2026.100896)
Supplement: Supplementary file 6 [file mmc6.docx]

| Vaccination Status | PCR result | | | | | | | |
| --- | --- | --- | --- | --- | --- | --- | --- | --- |
|  | Negative | | Positive | | Not Done | | Total | |
|  | N | % | n | % | n | % | N | % |
| Ever Been Vaccinated to Bacterial Meningitis | 166 | 17.8 | 32 | 0.1 | 0 | 0.0 | 198 | 21.2 |
| **Meningococcal Vaccine** | | | | | | | | |
| Yes* | 11 | 1.4 | 0 | 0.0 | 0 | 0.0 | 11 | 1.2 |
| *MenACWY(Conjugate)* | *11* | *100.0* | *0* | *0.0* | *0* | *0.0* | *11* | *100.0* |
| No | 442 | 54.6 | 83 | 68.6 | 0 | 0.0 | 522 | 55.9 |
| Unknown ** | 357 | 44.1 | 37 | 30.6 | 2 | 100.0 | 396 | 42.4 |
| **Pneumococcal Conjugate Vaccine** | | | | | | | | |
| Yes* | 14 | 1.7 | 2 | 1.7 | 0 | 0.0 | 16 | 1.7 |
| *23-valent polysaccharide vaccine (PPSV23)* | *1* | *7.1* | *0* | *0.0* | *0* | *0.0* | *1* | *6.3* |
| *10-valent pneumococcal conjugate (PCV10)* | *11* | *78.6* | *1* | *50.0* | *0* | *0.0* | *12* | *75.0* |
| *13-valent pneumococcal conjugate (PCV13)* | *1* | *7.1* | *0* | *0.0* | *0* | *0.0* | *1* | *6.3* |
| *Not reported* | *1* | *7.1* | *1* | *50.0* | *0* | *0.0* | *2* | *12.5* |
| No | 431 | 53.2 | 84 | 69.4 | 0 | 0.0 | 515 | 55.2 |
| Unknown ** | 365 | 45.1 | 35 | 28.9 | 2 | 100.0 | 402 | 43.1 |
| **Haemophilus influenzae type b** | | | | | | | | |
| Yes | 141 | 17.4 | 30 | 24.8 | 0 | 0.0 | 171 | 18.3 |
| No | 333 | 41.1 | 60 | 49.6 | 0 | 0.0 | 393 | 42.1 |
| Unknown ** | 336 | 41.5 | 31 | 25.6 | 2 | 100.0 | 369 | 39.5 |
| *Rates are based on having the denominator (total) equal to those who received the vaccine type  ** Unknown cases are derived from the self-reported information provided by the patients or their guardians. | | | | | | | | |
