# Supplementary material for: Strengthening regional surveillance: MenMap Network’s year 1 findings on bacterial meningitis in Jordan, Egypt, and Iraq (2023-2024)
Source: IJID Reg. 2026 Apr 16;19:100896. doi: 10.1016/j.ijregi.2026.100896 (PMC13147366; doi:10.1016/j.ijregi.2026.100896)
Supplement: Supplementary file 7 [file mmc7.docx]

| **Sign and Symptoms** | **Country** | | | | | | | |
| --- | --- | --- | --- | --- | --- | --- | --- | --- |
|  | **Regional** | | **Jordan** | | **Egypt** | | **Iraq** | |
|  | **N** | **%** | **n** | **%** | **n** | **%** | **n** | **%** |
| Fever | 180 | 94.2 | 27 | 100.0 | 43 | 100.0 | 110 | 90.9 |
| Vomiting | 97 | 50.8 | 11 | 40.7 | 24 | 55.8 | 62 | 51.2 |
| Headache | 78 | 40.8 | 2 | 7.4 | 27 | 62.8 | 49 | 40.5 |
| Neck Stiffness | 75 | 39.3 | 0 | 0.0 | 20 | 46.5 | 55 | 45.5 |
| Seizures | 66 | 34.6 | 4 | 14.8 | 13 | 30.2 | 49 | 40.5 |
| Lethargy | 62 | 32.4 | 9 | 33.3 | 1 | 2.3 | 52 | 43.0 |
| Altered consciousness | 40 | 20.9 | 2 | 7.4 | 21 | 48.8 | 17 | 14.0 |
| Irritability | 38 | 19.9 | 5 | 18.5 | 7 | 16.3 | 26 | 21.5 |
| Photophobia | 31 | 16.2 | 1 | 3.7 | 0 | 0.0 | 30 | 24.8 |
| Nausea | 25 | 13.1 | 1 | 3.7 | 0 | 0.0 | 24 | 19.8 |
| Diarrhea | 22 | 11.5 | 3 | 11.1 | 0 | 0.0 | 19 | 15.7 |
| Abdominal pain | 19 | 8.9 | 0 | 0.0 | 2 | 4.7 | 15 | 12.4 |
| Bulging fontanels | 16 | 8.4 | 1 | 3.7 | 2 | 4.7 | 13 | 10.7 |
| Leg pain | 9 | 4.7 | 0 | 0.0 | 2 | 4.7 | 7 | 5.8 |
| Chills | 8 | 4.2 | 0 | 0.0 | 0 | 0.0 | 8 | 6.6 |
| Toxic appearance | 6 | 3.1 | 1 | 3.7 | 0 | 0.0 | 5 | 4.1 |
| Myalgia | 5 | 2.6 | 0 | 0.0 | 0 | 0.0 | 5 | 4.1 |
| Cold extremities | 4 | 2.1 | 1 | 3.7 | 0 | 0.0 | 3 | 2.5 |
| Purpuric / hemorrhagic skin lesion | 3 | 1.6 | 0 | 0.0 | 0 | 0.0 | 3 | 2.5 |
| Rash | 3 | 1.6 | 1 | 3.7 | 0 | 0.0 | 2 | 1.7 |
| Characteristic purpura that does not blanch on pressure | 2 | 1.1 | 0 | 0.0 | 0 | 0.0 | 2 | 1.7 |
| Delay in capillary refill time | 1 | 0.5 | 0 | 0.0 | 0 | 0.0 | 1 | 0.8 |
