# Supplementary material for: Strengthening regional surveillance: MenMap Network’s year 1 findings on bacterial meningitis in Jordan, Egypt, and Iraq (2023-2024)
Source: IJID Reg. 2026 Apr 16;19:100896. doi: 10.1016/j.ijregi.2026.100896 (PMC13147366; doi:10.1016/j.ijregi.2026.100896)
Supplement: Supplementary file 8 [file mmc8.docx]

| **Antibiotic Type** | **Country** | | | | | | | |
| --- | --- | --- | --- | --- | --- | --- | --- | --- |
|  | **Regional** | | **Jordan** | | **Egypt** | | **Iraq** | |
|  | **N** | **%** | **n** | **%** | **n** | **%** | **n** | **%** |
| Amoxicillin | 2 | 1.1 | 1 | 3.7 | 0 | 0.0 | 1 | 0.8 |
| Ceftriaxone | 179 | 93.7 | 23 | 85.2 | 43 | 100.0 | 113 | 93.4 |
| Penicillin G | 0 | 0.0 | 0 | 0.0 | 0 | 0.0 | 0 | 0.0 |
| Vancomycin | 103 | 53.9 | 7 | 25.9 | 5 | 11.6 | 91 | 75.2 |
| Oxacillin | 0 | 0.0 | 0 | 0.0 | 0 | 0.0 | 0 | 0.0 |
| Unknown* | 0 | 0.0 | 0 | 0.0 | 0 | 0.0 | 0 | 0.0 |
| Other** | 40 | 20.9 | 4 | 14.8 | 4 | 9.3 | 32 | 26.4 |
| *Cases where the type of antibiotic treatment was not recorded or documented in the dataset.  **This category groups less frequently reported antibiotics that were not part of the main list. | | | | | | | | |
